# An Assessment of Knowledge, Attitude, and Practices of Birth and Death Registration in Kilifi County in the Coastal Region in Kenya

**DOI:** 10.1155/2021/9963703

**Published:** 2021-10-01

**Authors:** Samwel Wakibi, Ezekiel Ngure

**Affiliations:** University of Nairobi, Kenya

## Abstract

**Background:**

Countries need vital statistics for social and economic planning. World Health Organization (WHO) recommends at least 80% coverage to use registration data on births and deaths for social and economic planning. However, registration remains low in developing countries. National coverage for Kenya in 2014 was 62.2% for births and 45.7% for deaths, with wide regional differentials. Kilifi County in the coastal region in Kenya reported rates below the national coverage at 56% for births and 41% for deaths in 2013.

**Objective:**

To determine level of knowledge and practice and reasons for low coverage of birth and death in Kilifi County.

**Method:**

This is a descriptive cross-sectional study that employed multistage cluster random sampling procedure to select a sample of 420 households from which household heads and women with children below five years old were surveyed.

**Results:**

Out of the 420 households sampled, about all respondents (99%) were aware of birth registration while death was 77%. Their main sources of information were assistant chiefs at 77% for both birth and death registration and family and friends at 67% for deaths and 52% for births. Coverage for birth registration was 85% and death 63%. More deaths occurred at home (55%) than in hospital (44%) while 55% of deliveries occurred in hospital and 44% at home. Main reasons for not registering death were ignorance (77%) and transport and opportunity cost (21%) while for birth registration were ignorance (42%), travel and opportunity cost (41%), lack of identification documents (9%), and home deliveries (7%).

**Conclusion:**

Registration of birth and death has improved in Kilifi County. The drivers are legal and requirements to access social rights. Reasons for not registering are ignorance and opportunity costs. Community should be sensitized on the importance of registration, address home deliveries and deaths, and increase efficiency in registration. Further research is recommended to determine the severity of teenage pregnancy and orphanhood in the county.

## 1. Introduction

Vital statistics are necessary for determining population changes, public administration, policy formulation, planning, and implementation of development policies. Ideally, birth registration is part of an effective civil registration system that acknowledges the existence of the person before the law, establishes the child's family ties, and tracks the major events of an individual's life, from live birth to marriage and death. A birth certificate provides some, albeit minimal protection against early marriage, child labour, recruitment in the armed forces, or detention and prosecution as an adult [1]. The data are required to formulate programs relating to maternal and child health including nutrition, immunization, and universal education.

World Health Organization (WHO) recommends at least 80% coverage, as criteria for use of registration data on births and deaths. However, coverage of birth and death registration remains unacceptably low especially in developing countries.

Globally, each year, about two-thirds of 57 million annual deaths go unregistered, and as much as 40% (48 million) of 128 million births go unregistered, representing one out of three children [2]. Although it can be argued that census and other large sample surveys may be useful in supplementing demographic data in countries where vital registration system is still at infancy, they are expensive to perform on a routine basis, are frequently marred by politics, disputes about figures, underfunding, and topographical challenges, and should rather serve as complements in a comprehensive health information system [3, 4].

Civil registration of vital events in Kenya started in 1904, but was limited to Europeans and Americans. However, after independence in 1963, registration was made compulsory for all residents in Kenya. The Civil Registration Service (CRS) is the government agency responsible for the registration of births and deaths. The assistant chiefs are the government registration agents for vital events that occur at home or in the community while health care workers are responsible for events that occur in health institutions. The agents submit notifications to civil registrars in civil registration offices for registration and issuance of birth and death certificates.

Despite the clear path, registration coverage for Kenya is below WHO recommended levels. The national coverage was 62.2% for births and 45.7% for deaths in 2014, with wide regional differentials which suggests that factors determining coverage may vary by county. However, very few community studies have been conducted in Kenya to determine the factors responsible for the low coverage. This study was undertaken to identify factors responsible for low registration of birth and death in Kilifi County; the coverage was below the national coverage at 56 and 41 percent, respectively, in 2013. The study assessed knowledge, attitude, and practice (KAP) of birth and death registration in Kilifi County.

## 2. Materials and Methods

### 2.1. Study Site

The study was conducted in Kilifi County. The constitution of Kenya divides the territory of Kenya into 47 geographical units, and Kilifi County is one of the units. Kilifi County is located in the coastal region in Kenya and has an area of 12,245 km^2^. According to Kenya national population census, the county's population was 1,109,735 in 2009 [5] with a growth rate of 3.1 percent per annum. The main economic activities are tourism and fishing due to its proximity to the Indian Ocean. It has fertile soils and good weather pattern, and so, it is good for agricultural farming.

### 2.2. Sampling and Sample Size

This study employed a multistage cluster random sampling procedure. A sample of 420 households was drawn from twelve (12) sublocations selected from four subcounties. The four subcounties were randomly selected from the six subcounties that make up Kilifi County. Thirty-five (35) households were selected systematically from each sublocation.

The main tool for the study is survey questionnaire—a household questionnaire with vital event sections for deaths in the last five years to the survey and births less than five (5) years old. Interview guides were also utilized to collect qualitative data to explain the survey findings.

### 2.3. Study Design and Target Population

The KAP survey was cross-sectional and targeted household heads and women with children below five (5) years old. The respondents were interviewed to elicit information on household characteristics, registration of deaths that occurred in the last five years, and births below five years old. The targeted women also provided information on their experience with the civil registration system (recent/current bottlenecks in civil registration of births) and reasons for not registering birth.

To address known limitations of quantitative design, qualitative techniques (KII and FGD) were employed to contextualize and supplement the survey findings and explain the practices and attitudes in survey questionnaire responses. The qualitative study targeted registration agents to understand the CRS system and community elders to understand the sociocultural context. The qualitative interviews were aimed at describing and understanding the community's own perceptions and experience in birth and death registration [6].

## 3. Results

To achieve the study objectives, 420 household were surveyed, and six focus group discussions were held with members of the community and seven key informant interviews with birth and death registration agents.

From Table 1, out of the 420 households sampled, 88 (21%) were in urban and 332 (79%) in rural area. Over 10 and 13 percent of households surveyed were more than 10 km away from the assistant chief's office and health facility (registration agents), respectively. About 84.3 percent of the households had at least one child under the age of five (5) years, while more than a quarter (27%) of households had experienced death in the last five (5) years.

Of the 420 respondents to the household questionnaire, 164 (39%) were heads of household and 237 (56%) were spouses of the heads of household. Two hundred and sixty-four (63%) of the respondents were female while 37 percent were male. Over 80 percent of respondents were between 25 and 49 years old, about 10 percent were 50 or more years old, while less than nine percent (8.8%) were between 20 and 24 years old. Thirty percent (30%) of respondents had no formal education, 55 percent had some primary education, less than 14 percent (13.9%), and less than 2% had some secondary and tertiary education, respectively (Table 2).

More than three quarters (76%) of respondents were from monogamous families, less than 17 percent were polygamous, 4 percent had never married, while about 3 percent were widowed, divorced, or separated. The median household size was 7 persons. Two hundred and eighty-four (68%) of the respondents had children below 5 years (Table 2).

Out of the 420 respondents interviewed, all respondents (99.5%) were aware of birth registration, while more than three quarters (322 (76.7%)) were aware of death registration. Their main sources of information on death registration were assistant chiefs (77%) and family and friends (67%). Main source of information for birth registration was also the assistant chief (77.2%) and members of the family and friends (51.7%). However, only 11 percent of respondents reported having heard of death registration from health workers compared to 37.8 percent for birth registration (Table 3).

Almost all (97%) of the 322 respondents and of the 419 respondents who said they were aware of death and birth registration, respectively, had knowledge of at least one place where to register the respective civil event. Among the respondents aware of birth registration, higher percentage (94%) of them knew how to register birth compared to respondents who were aware of death registration where only 69 percent were knowledgeable on the process of registering death.

Majority (66%) of the respondents who were aware of death registration were also aware of importance of death registration. Fifty-nine (59) cited legal requirement and 2 percent to obtain a burial permit. A sizable percentage mentioned individual benefits: 37 percent for succession and 14 percent to honour the deceased. However, 47 (11%) respondents had no idea why registration of death is done.

For birth registration, almost all (93%) of the 419 respondents who were aware of birth registration were also aware of the importance of registering birth. The reasons advanced for registering birth varied from to meet legal requirement (40%), school requirement to register for national examinations and to access bursaries for orphans (71%), to acquire national identification card (ID) and passport (36.4%), and because it is good to obtain a birth certificate for any eventuality (17.5%). However, 28(6.7%) of the respondents had no reason why registration should be registered (Table 3).

The survey reported 140 deaths and 671 births in the last five years prior to the survey. Out of the 140 deaths, 79 (56%) were male and 61 (44%) female, and more deaths occurred at home (55%) than in hospital (44%). For births, about 333 (50%) were male and 338 female (50%) and were more hospital deliveries (55%) than home deliveries (44%). Of the 671 birth reported, 568 (85%) were registered but only 90 (13%) had been issued with a birth certificate. As regards death, out of the 140 deaths reported, 118 had been registered but only 27 (19%) had been issued with death certificate (Table 4).

Reasons for not registering death are ignorance (54%: did know where to register and did not know the importance), not heard of death registration (23%), distance to registration office, long wait in the queue and costs associated with travel (21%), and deaths that occurred at home (9%). For birth registration, reasons for not registering are ignorance (42%), transport and associated costs (21%), distance to registration office and waiting time (20%), lack of identification documents (9%), and home deliveries (7%).

## 4. Discussions

### 4.1. Knowledge of Civil Registration System

Knowledge about death registration in Kilifi County is high at 77% but is lower than birth registration (99%). This can be explained by the number of interventions in Kilifi County; most CRS partners are implementing interventions addressing issues of late and low registration coverage for births while there is none for death registration. The main driver for death registration in the region is the legal requirement for burial permit for body disposal and succession.

The low level of awareness found can be associated with low level of education among residents in this area; only about 15 percent of the respondents have attended school beyond primary level with 30 percent with no formal education. Other similar studies have found that where awareness about registration of civil events is low, coverage is also low [3, 7]. According to UNICEF, unregistered children tend to be found in areas where there is little awareness of the value of birth registration [8].

The respondents found in this study to be ignorant about civil registration especially death registration and about place to register birth and death, have no individual or legal incentives to register, and are not clear about the process of registering are therefore unlikely to register birth and death if and when it occurs in their households. This shows that awareness about civil registration among the people is one of the important reasons for low coverage of birth and death registration in Kilifi County.

The study also established that the main sources of knowledge on birth and death registration are the registration agents, family, and relatives. The role of media in this regard is minimal. This is not surprising as the study found most (84.5%) of the respondents had not attended school beyond primary level and therefore not amenable to source of information outside of their social circles. Any awareness campaign on civil registration targeting the community should therefore be through communal activities including meetings, wedding, and funerals.

### 4.2. Practice

Crude birth rate (CBR) and crude death rate (CDR) are indicators of levels of living or quality of life [9]. In this study, crude birth and death rates for Kilifi County were 38.9 and 8.3 per 1000 population, respectively. The CBR compared well with that reported in WorldBank report 37.6 per 1000 population in the year 2011; however, the CDR reported was in variance to the WorldBank's 11.8 per 1000 population in the year 2011. This may be attributed to under reporting of deaths especially for neonates which are regarded as bad omen and are interred immediately after death as explained by a respondent in FGDs: *Stillbirths and deaths of newborn babies are mostly not reported, especially when they take place at home. They are buried immediately due to cultural beliefs- they are a bad omen.... Sometimes back they used to be buried under the bed or under a big tree and the whole thing (death) is forgotten.*

For a CDR of 11 per 1000 population [10, 11] and a sample size of 3360 (420 households and household size of 8) people represented in this study, expected number of deaths is 37 per year or 185 deaths in five years as opposed to 140 deaths reported in this study. The 45 people whose deaths (185 deaths computed—140 deaths reported) were not reported might have suffered the “scandal of invisibility” [12].

The study found coverage of birth and death registration in Kilifi to be 85 percent and 84 percent, respectively. However, the death coverage is in variance with WorldBank 63.3 percent (computed based on WorldBank CDR of 0.011) which can be attributed to low death reporting implied elsewhere in this report.

Birth registration was found to vary by place of residence, age of the mother, marital status, and education level of household head. Birth registration is highest among children in urban areas than rural areas which can be attributed to strong links to the mainstream mechanisms of society, such as health services. About 60 percent of births in urban areas occurred in hospitals compared to 54 percent in rural areas. Kumar et al. reported similar finding in Eastern Uttar Pradesh where birth coverage varied by place of residence with urban area reporting better coverage than rural area, parent's level of education, and social economic status and marital status of the mother [13].

However, the study found low uptake of event certificate: 19 and 13 percent for death and birth, respectively. This can be interpreted as low individual incentive for death and birth certificates in the area. UNICEF reported similar findings where 85 percent of registered children under the age of five did not have birth certificate [8]. The little demand for birth and death certificate can be explained by the low level of awareness of their importance reported in this study.

The proportion of deaths with death certificate is higher than for birth certificates. This maybe because of succession and inheritance which is usually shortly after death, but unlike birth certificate which will be required when the children will be joining school at age six or seven.

The birth and death registration rates reported in this study are too high compared to 56% and 41.1 for birth and death, respectively, reported in the annual vital statistics of 2013. The striking gap between the rate of death registration in the study and the vital statistics published in 2010-2013 can be explained by under reporting of deaths reported in the study or speculate loss of forms/data between the agent and CRO office.

### 4.3. Reasons Advanced for Birth and Death Registration

Drivers of registration of deaths and births in this area are as follows: the need to satisfy legal requirement, to meet school and bursary requirement, to acquire passport and ID, and succession. The perception is on the need for a certificate to achieve something else [3, 14]. For example, the actual death registration is highly driven by the legal requirement to obtain a burial permit for disposal of the body while for birth registration, the key motivating factor is the requirement for birth certificate to access social rights including education in future [3]. This indicates that to increase birth and death registration in this area, appropriate incentive(s) are required; lack of sufficient incentives or pressures on the citizen to register leads to low coverage [7].

Reasons advanced by respondents for not registering vital events and, where registered, reason for not obtaining birth and death certificate were distance to the registration office and associated costs, waiting time and opportunity cost for gainful work, and ignorance—not knowing the importance of birth and death registration [7, 13].

### 4.4. Challenges in Birth and Death Registration

The death and birth registration system faces various challenges that affect its optimal performance especially completeness and data quality. The issues include the following:

- *Shortage of Registration Materials*. An assistance chief explained that sometimes he is forced to record details of a vital event that is reported in a note book and transfer them to the notification forms when supplies are replenished.
- *Competing Priorities*. Registration of vital events is not always a priority among registration agents work—it is secondary to other tasks performed by the registration agents including clinical work among health workers and public administration duties for assistant chiefs.
- *Limited Knowledge on Event Registration among Agents*. It was established that some agents especially chiefs are asking for more information than is necessary (details of the child's father) as a requirement to register a birth, while some agents in health facilities do not understand their role as registration agents (a nurse refusing to fill in notification form). The agents should be sensitized on death and birth registration especially on the requirements for event registration.
- *Low Staffing Leading to Long Queues and Waiting Time* [8]. Kilifi County has two (2) CROs serving a population of 1,109,735 (Census 2009) with a growth rate of 3.1 percent per annum and an area of 12,245 km^2^. Countries where CRVS is fully developed, in average, a civil registration unit serves between 10,000 and 34,000 population within a much smaller geographical area.
- *Incorrect Certification of Births/Falsification of Records by Residents*. Respondents reported rampant use of names and ID of relatives inappropriately to register births. This is meant to cheat the system that requires details of the child's father and identification card.

## 5. Conclusion

Death and birth registration in Kilifi County has improved. However, gaps in awareness, lack of clarity about the registration process, and individual perceptions are contributory factors to suboptimal civil registration in Kilifi.

Leading reasons for not registering are distance, long queues/overnight stay for the services, and associated costs. Others are ignorance: never heard of civil registration, do not know the importance and process of registration, and inertia—too many births and deaths occurring at home.

Drivers of registration are legal requirements and requirement to access social rights including education. Reasons for not registering are ignorance, opportunity and travel cost, and death and delivery occurring at home.

Generally, respondents perceived registration of births and deaths as expensive both in terms of travel and opportunity cost, and it has little or no immediate benefits to the individuals and thus not a priority.

To improve coverage for both births and deaths, the study recommends the following:

- Enhance awareness campaign among residents on civil registration in the area. The most effective channels of awareness creation are community meetings including, weddings, burials, and community health workers (CHW). The messages should include registration procedure, place to register, and importance of birth and death registration as incentives
- Extend birth and death registration network to subcounty level by either opening registration offices or introducing mobile birth and death registration services. This will reduce distance residents have to travel to access registration services, consequently cut cost of transport and associated expenses, and reduce time away from daily work
- Enforce the law on birth and death registration
- Avoid stock out of registration materials—application forms
- Undertake a validation exercise/study to ascertain system efficiency in particular the link between the registration agents and Civil Registration Offices

## Figures and Tables

**Table 1:** Household characteristics and relationship of respondents to head of household.

Place of residence	Household head	Spouse	Other relatives	Household median	Size mean	Total (*N*)
Urban	30.7%	67.0%	2.3%	7	10.8	88 (21%)
Rural	41.3%	53.6%	5.1%	8	10.8	332 (79%)
Total	39.0%	56.4%	4.6%	8	10.8	420

Households characteristics			Urban %	Rural %	*N* (%)	
Children < 5 yrs:						
(i) Yes			80.7%	85.5%	354 (84.3)	
(ii) No			19.3%	14.5%	66 (15.7)	
Household reported having death in the last 5 years:						
(i) Yes			29.5%	27.8%	119 (28.3)	
(ii) No			70.5%	72.2%	301 (71.7)	
Distance to registration agent (assistant chief):						
(i) Less than 10 kilometers			98.9%	86.4%	372 (89)	
(ii) More than 10 kilometers			1.1%	13.6%	46 (11)	
Distance to registration agents (health facility):						
(i) Less than 10 kilometers			100%	83.9%	365 (87.3)	
(ii) More than 10 kilometers			0%	16.1%	53 (12.7)	

**Table 2:** Background characteristics of respondents.

Respondents characteristics	*N* (%)	Respondents characteristics	*N* (%)
Age:		Level of education:	
Under 20	4 (1%)	No school	124 (29.6%)
20-24	37 (8.8%)	Primary not completed	147 (35.1)
25-34	124 (29.5)	Primary completed	83 (19.8)
35-49	214 (51)	Secondary uncompleted	23 (5.5)
Over 49	41 (9.8)	Secondary completed	35 (8.4)
Sex:		Higher	7 (1.7)
Male	156 (37.1)	Years lived in Kilifi:	
Female	264 (62.9)	Less than 5 years	18 (1.8)
Marital status:		More than 5 years	402 (98.2)
Never married	17 (4%)	Age of last born child	
Married—monogamous	320 (76.2)	Less than 5 years	284 (68.1)
Married—polygamous	70 (16.7)	5-19 years	94 (22.5)
Divorced/separated	3 (0.7)	More than 20 years	37 (8.9)
Widowed	10 (2.4)	No child	2 (0.5)

**Table 3:** Community knowledge, attitude and practice of birth and death registration.

Births	*N* (%)	Deaths	*N* (%)
Heard of registration of birth?		Heard of registration of death?	
(i) Yes	418 (99.5)	(i) Yes	322 (76.7)
(ii) No	2 (0.5)	(ii) No	98 (23.3)

Source of information about birth registration: (multiple response)	*N* = 418	Source of information about death registration: (multiple response)	*N* = 322
(i) Family and friends	216 (51.7)	(i) Family and friends	217 (67)
(ii) Mass media	16 (3.8)	(ii) Mass media	22 (7)
(iii) Health worker	158 (37.8)	(iii) Chief or assistant chief	247 (77)
(iv) Assistant chief	323 (77.2)	(iv) Health worker	36 (11)

Place to register: (multiple response)	*N* = 418	Place to register: (multiple response)	*N* = 322
(i) Hospital	208 (50%)	(i) Hospital	48 (15)
(ii) Chief's office	335 (80.1)	(ii) Chief's office	295 (92)
(iii) Registrar's office	62 (14.8)	(iii) Registrar's office	32 (10)
(iv) Do not know	10 (2.4)	(iv) Do not know	9 (3)

How to register:	*N* = 417	How to register:	*N* = 322
(i) Birth certificate	70 (16.7)	(i) Death certificate	33 (10)
(ii) Notification only	323 (77)	(ii) Notification only	257 (80)
(iii) DK/cannot remember	24 (5.7)	(iii) DK/cannot remember	32 (10)

Knowledge Level	*N* = 415	Knowledge Level	*N* = 321
(i) Adequate	234 (56)	(i) Adequate	207 (64)
(ii) Inadequate	181 (44)	(ii) Inadequate	114 (36)

In your opinion, is birth registration necessary?	*N* = 420	In your opinion, is birth registration necessary?	*N* = 420
(i) Yes	390 (93)	(i) Yes	275 (65.5)
(ii) No	28 (6.5)	(ii) No	47 (11)
(iii) Not heard of birth registration	2 (0.5)	(iii) Not heard of birth registration	98 (23.5)

Reason for registering births (opinion): (multiple response)	*N* = 418	Reason for registering deaths (opinion): (multiple response)	*N* = 322
(i) Legal requirement	161 (38.5)	(i) Government requirement	190 (59)
(ii) School and bursary	298 (71.3)	(ii) Succession	118 (37)
(iii) To acquire passport and ID	152 (36.4)	(iii) To honour/remember deceased	45 (14)
(iv) Good to have/feeling	73 (17.5)	(iv) Burial permit	8 (2)
(v) Do not know	28 (6.7)	(v) Do not know	47 (15)
		(vi) Sponsorship and ID	3 (1)

Reasons for not registering births (opinion): (multiple response)		Reasons for not registering deaths (opinion): (multiple response)	*N* = 420
(i) Cultural and religious beliefs	5 (0.9%)	(i) Cultural and religion beliefs	12 (3)
(ii) Transport and associated cost	120 (20.8)	(ii) Distance, waiting time, and associated costs	50 (21)
(iii) Distance to registration agents and waiting time	115 (19.9)	(iii) Death occurring at home	38 (9)
(iv) Not having ID	54 (9.4)	(iv) Do not know importance	225 (54)
(v) Ignorance (do not know importance)	244 (42.3)	(v) Not heard of death registration	98 (23)
(vi) Home deliveries	39 (6.8%)	(vi) Others (ID, documents lost)	6 (1)

**Table 4:** Characteristics of births and deaths in the last five years and reason for not registering.

Birth	*N* (%)		*N* (%)
Sex:		Place of birth:	
(i) Male	333 (49.6)	(i) Hospital	374 (55.4)
(ii) Female	338 (50.4)	(ii) Not in hospital	293 (44)
(iii) Total	671	(iii) Do not know	4 (0.6)
Registered/notified:		Certificate issued:	
(i) Yes	568 (84.6)	(i) Yes	90 (13.4)
(ii) No	34 (5.1)	(ii) No	575 (85.7)
(iii) DK	69 (10.3)	(iii) DK	6 (1)
Death	*N* (%)		*N* (%)
Sex:		Place of death:	
(i) Male	79 (56.4)	(i) Hospital	61 (43.6)
(ii) Female	61 (43.6)	(ii) Not in hospital	77 (55)
Registered/notified:		Certificate issued:	
(i) Yes	118 (84.3)	(i) Yes	27 (19.3)
(ii) No.	4 (2.9)	(ii) No.	108 (77.1)
(iii) DK	18 (12.9)	(iii) DK	5 (3.6)
Reason for not registering death:			
(i) Lack of funds			
(ii) Not knowing place to register			
(iii) Ignorance (lack of knowledge, stillbirth, did not know the importance)			

## Data Availability

Data is available on request.

## Additional Points

*Paper Context*. Birth and death registration in Kilifi County in Kenya is below national coverage. Reasons for suboptimal coverage are not known. This study has identified the reasons and recommended measures to address them. If the measures are implemented, coverage would improve to more than 80% and the data from the civil registration system can therefore be used for social and economic planning in Kenya.
